# Cardiac findings on Non Cardiac Computed Tomography

**DOI:** 10.5334/jbr-btr.1036

**Published:** 2016-02-04

**Authors:** Jean-Nicolas Dacher

**Affiliations:** 1 CHU Rouen, FR

The heart has long been ignored on chest computed tomography (CT), often neglected by the radiologist and considered as a blind moving structure optimally investigated by echocardiography. With the improving technology including multiple detectors, high-speed rotation and increased spatial resolution, many cardiac diseases can now be identified on chest CT, even without ECG-gating.

The most straightforward diagnosis is calcification. The presence of coronary artery calcification is a marker of coronary artery disease. It can orient the patient towards further investigation, or at least medical prevention. Pericardial calcification should be mentioned even if subtle, since it could indicate constriction. Aortic or mitral valve calcifications deserve mention as well. Aortic valve calcification correlates with the degree of stenosis. Both are markers of an increased risk of cardiovascular accident.

Thrombus is frequently encountered on chest CT; it should be reported due to the high risk of migration and embolus. In the left ventricle, the most common location is the apex following anteroseptal myocardial infarction, which may be occult. In the elderly, thrombus of the left atrial appendage (LAA) is a frequent incidental finding carrying a high risk of stroke. Decreased attenuation of the LAA can be due to stagnant blood (in cases of dilated left atrium and/or atrial fibrillation) or thrombus, and the differential diagnosis is made by a five-minute delayed scan.

The most commonly found tumour is myxoma, usually a huge mass located in the left atrium, attached by a thin pedicle to the foramen ovale.

In the context of acute chest pain, CT is frequently performed on an emergency basis to rule out aortic dissection or pulmonary embolus. In the absence of such anomalies and when few motion artefacts are present (the *still heart* sign), it is of importance to have a close look at the myocardium. It is not infrequent to identify myocardial infarction appearing as an area of decreased attenuation on arterial phase.

Left ventricular diameter, myocardial thickness and trabeculation should also be examined, for detecting hypertrophic, dilated or non-compaction cardiomyopathy.

Finally, it is important to detect and report any anomaly in size of the ascending aorta; aneurysm of the ascending aorta is a frequent incidental finding carrying a size-dependent risk of complications.

## Competing Interests

The author declares that they have no competing interests.

